# Simple synthesis of highly catalytic carbon-free MnCo_2_O_4_@Ni as an oxygen electrode for rechargeable Li–O_2_ batteries with long-term stability

**DOI:** 10.1038/srep13266

**Published:** 2015-08-21

**Authors:** Ramchandra S. Kalubarme, Harsharaj S. Jadhav, Duc Tung Ngo, Ga-Eun Park, John G. Fisher, Yun-Il Choi, Won-Hee Ryu, Chan-Jin Park

**Affiliations:** 1 Department of Material Science and Engineering, Chonnam National University, 77, Yongbongro, Bukgu, Gwangju 500-757, South Korea; 2 Central Research Center, Doosan Heavy Industries & Construction Co., 22, Doosan volvoro, Seongsangu, Changwon 642-792, Gyeongsangnamdo, South Korea; 3 Department of Chemical and Environmental Engineering, Yale University, New Haven, Connecticut 06520-8286, United States

## Abstract

An effective integrated design with a free standing and carbon-free architecture of spinel MnCo_2_O_4_ oxide prepared using facile and cost effective hydrothermal method as the oxygen electrode for the Li–O_2_ battery, is introduced to avoid the parasitic reactions of carbon and binder with discharge products and reaction intermediates, respectively. The highly porous structure of the electrode allows the electrolyte and oxygen to diffuse effectively into the catalytically active sites and hence improve the cell performance. The amorphous Li_2_O_2_ will then precipitate and decompose on the surface of free-standing catalyst nanorods. Electrochemical examination demonstrates that the free-standing electrode without carbon support gives the highest specific capacity and the minimum capacity fading among the rechargeable Li–O_2_ batteries tested. The Li-O_2_ cell has demonstrated a cyclability of 119 cycles while maintaining a moderate specific capacity of 1000 mAh g^−1^. Furthermore, the synergistic effect of the fast kinetics of electron transport provided by the free-standing structure and the high electro-catalytic activity of the spinel oxide enables excellent performance of the oxygen electrode for Li-O_2_ cells.

The increase in energy demand as a result of increased living standards and population has motivated the efforts to develop high energy density power sources. Even though incremental improvements have been made in Li-ion battery technology, this advancement has not kept pace with the development of portable devices, leaving a so-called “power gap” that is broadly anticipated to grow in coming years. Nevertheless, even when fully developed, the highest energy densities Li-ion batteries are unable to deliver sufficient energy to meet the demands of key markets such as transportation in the long term. In this regard, reaching beyond Li-ion batteries appears to be a formidable challenge; it requires the exploration of new materials and new chemistries, especially electrochemistries. As an alternative, lithium-air (lithium-oxygen) and lithium-sulfur batteries are gaining much attention due to their high theoretical specific energies, which are almost 6–10 times those of the state-of-the-art Li-ion batteries^1^,^2^,^3^. In particular, the lithium-oxygen (Li-O_2_) battery can use free oxygen from the air to react with lithium ions on the surface of the air (oxygen) electrode, which is much lighter than conventional cathodes used in Li-ion batteries. However, the critical challenges that limit the practical use of Li-O_2_ battery technology include the sluggish oxygen reduction reaction (ORR) during discharge and oxygen evolution reaction (OER) during charge in Li^+^-containing aprotic electrolytes.

In addition, the structure of the oxygen electrode also influences the performance of Li-O_2_ batteries. The essential prerequisite for the successful operation of a rechargeable Li-O_2_ battery is the formation of Li_2_O_2_ as a reaction product during discharge and the decomposition of Li_2_O_2_ to Li and O_2_ during charging. However, one of the critical problems in the non-aqueous Li-O_2_ batteries employing carbon based oxygen electrode is the very large polarization that occurs during the discharge/charge process. The high cell polarization is mainly attributed to the low catalytic activity of carbon and to the high activation energies required for the formation of Li_2_O_2_ during discharge and the decomposition of the Li_2_O_2_ during charging. It has been confirmed that the discharge/charge efficiencies can be improved by the addition of catalytic materials to the carbon supported oxygen electrodes. The catalysts included in the oxygen electrode can affect the discharge/charge potentials and determine the rechargeability of the cells. Furthermore, the extemporaneous parasitic reaction of carbon with Li_2_O_2_ is accountable for the formation of resistive carbonate at the interface between carbon and Li_2_O_2_ leading to large overpotentials during the OER and poor cycleability^4^,^5^. Furthermore, polyvinylidene fluoride (PVDF) used as a binder material in making the oxygen electrode can react with and be decomposed by oxygen radical intermediates to LiOH and LiF^6^,^7^. Therefore, it is vital to develop an effectively catalyzed oxygen electrode, a free-standing type, carbon and binder-free nanoarchitecture for the ORR and OER in Li-O_2_ batteries.

Thus far, several studies have reported the use of noble metals and metal oxides as a catalyst for Li-O_2_ batteries^8^,^9^,^10^,^11^,^12^. Transition metal oxides such as Co and Mn oxides have also been considered as potential candidate electrocatalysts for bi-functional oxygen electrodes due to their high catalytic activity and good corrosion stability for Li-O_2_ batteries^13^,^14^,^15^,^16^. Among various types of metal oxides, mixed transition metal oxides with a spinel structure are of interest as an electrocatalyst for ORR and OER due to their low cost, good stability, high activity, low toxicity, and simple preparation^17^,^18^,^19^,^20^. It is well known that the spinel compounds with general formula AB_2_O_4_ (A,B = Metal) are built around a closely packed array of O_2_^−^ ions, with A^2+^ and B^3+^ cations occupying part or all of the tetrahedral and octahedral sites, respectively. More importantly, in this structure the solid-state redox couples A^3+^/A^2+^ and B^3+^/B^2+^ are easily formed, which makes the spinel materials potentially suitable for electro-catalysis in OER and ORR. Recently, spinel materials anchored on carbon supports have been explored as an oxygen electrode material in Li-O_2_ batteries^20^,^21^,^22^. However, as mentioned above, the parasitic reaction of the carbon support with Li_2_O_2_ results in the limited cycle life for Li-O_2_ cells containing spinel catalysts^21^. Hence, to reduce side reactions due to carbon, a free-standing type oxygen electrode without carbon support or substrate needs to be developed and tested.

In the present work, we report a green, facile, and cost effective approach to engineer a free-standing type carbon and binder-free oxygen electrode. The hierarchical free-standing type spinel MnCo_2_O_4_ oxygen electrode developed in this study showed a reversible specific capacity of 10520 mAh·g^−1^ at an applied current of 100 mA·g^−1^ with the reduced charge-discharge potential gap of ~0.65 V. When the Li-O_2_ cell was cyclic tested using the limited capacity of 1000 mAh·g^−1^, the cycle life increased up to 119 cycles. The formation of a nano-porous architecture to accommodate discharge products and the increased electronic conductivity due to direct contact between the substrate and catalyst materials are the keys to improve the ORR and OER performance of the oxygen electrode and the associated cycle life of the Li-O_2_ cell. Herein, the main problem of low efficiency and poor cycle life caused by side reactions with the carbon support during the charge-discharge process in the oxygen electrode has been addressed.

## Results

**Figure 1:**
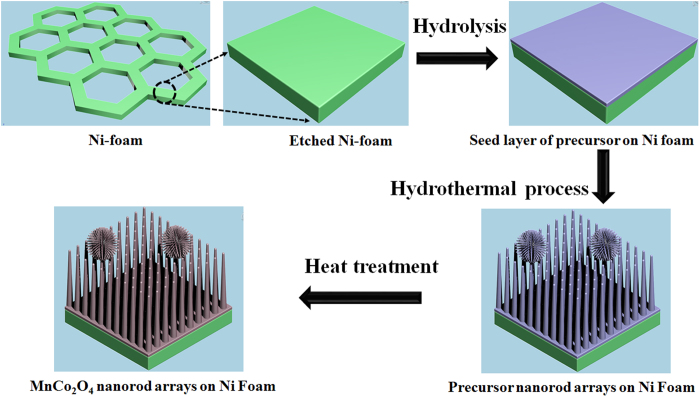
Schematic illustration of the growth mechanism for the free standing spinel MnCo_2_O_4_ nanorod arrays on Ni foam substrate as a carbon-free oxygen electrode for Li-O_2_ batteries.

**Figure 2:**
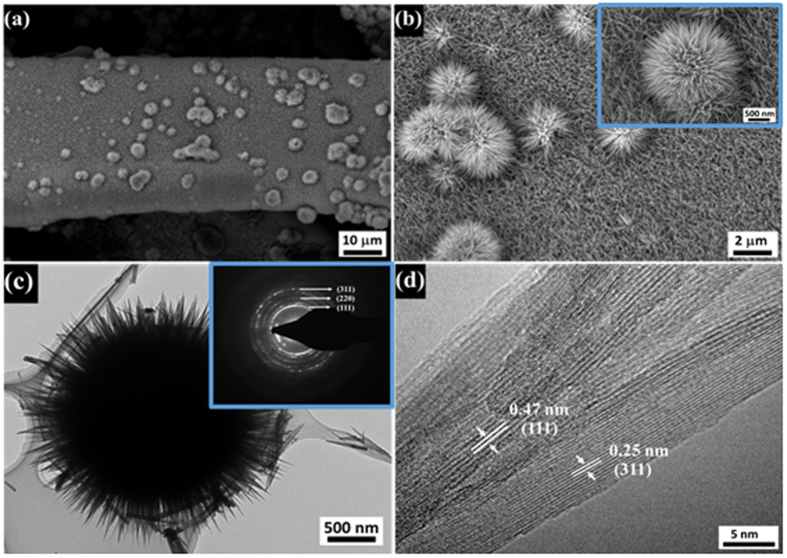
Microstructural exploration of the chestnut bur-like structures of MnCo_2_O_4_ formed on Ni foam. (**a**) low and (**b**) high magnification FE-SEM images; (**c**) TEM image and SAED pattern; (**d**) HR-TEM image.

Figure 1 schematically illustrates the strategy for the direct growth of ternary spinel MnCo_2_O_4_ nanorod arrays on metallic Ni foam substrates. In this process, the fresh Ni foam was slightly etched in an ultrasonic bath containing a mixture of HCl and HNO_3_ to remove the surface oxide layers and form fresh surface for the growth of spinel oxide. The etched Ni substrate was then immersed in the reaction solution containing Mn and Co precursors. During the hydrothermal process, at the initial temperature, the hydrolysis–precipitation process was initiated with the help of the reaction between urea and the metal ions leading to the formation of a thin seed layer of Mn, Co–hydroxide on the Ni substrate as shown in Supplementary Fig. S1. A very thin layer was observed on the nickel foam compared with the pristine Ni surface when removed from the solution after reaction for 1 h. The formed layer can act as the nucleation center for the growth of nanorod arrays. In the further hydrothermal process, the newly formed nuclei grew perpendicularly on the seed layer. As a result, large-scale, self-aligned Mn, Co–hydroxide nanorod arrays were formed on the conductive Ni foam. Some chestnut bur-like structures that might have grown from the pre-existing nanorod arrays were also formed, as confirmed in Fig. 2. The reactions for the formation of spinel MnCo_2_O_4_ are as follows.


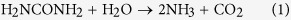



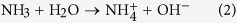



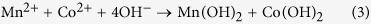



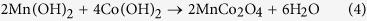


Thermo gravimetric analysis (TGA) of the powder, obtained by scratching the Ni-foam covered with the hydroxide precipitates, was carried out to determine the decomposition temperature of the precursor-hydroxides to form the spinel MnCo_2_O_4_ as given in Reaction 4. A typical TGA curve for the obtained powder is shown in Supplementary Fig. S2a. A weight loss of about 22.9 wt% was observed at 214–432 ^°^C through two steps, and is ascribed to the decomposition of the hydroxyl compounds in the precursor and the re-crystallization of the product after decomposition. According to the TGA curves, the sample appeared to be totally converted into the spinel phase at 432 °C, after which no further weight loss was observed, suggesting that pure spinel phase can be obtained by heat-treatment at temperatures higher than 432 °C. Hence, the materials grown on Ni foam after hydrothermal processing were annealed at 425 and 450 ^°^C for 2 h, respectively. The crystal structure of the formed material after annealing was confirmed by X-ray diffraction (XRD) analysis, and the results are presented in Supplementary Fig. S2b. The peaks indicated in the XRD pattern for the sample annealed at 450 ^°^C can be indexed to well-crystalline MnCo_2_O_4_ with the spinel structure (Fd-3 m). This structure is regarded as a mixed valence oxide exhibiting a cubic spinel structure in which manganese and cobalt are distributed over both octahedral and tetrahedral sites. Moreover, the sample annealed at 425 ^°^C was poorly crystalline, while the as-prepared sample showed peaks corresponding to the precursor hydroxides.

Figure 2a,b show SEM images of nanorod arrays of free-standing MnCo_2_O_4_ (FSMCO) formed on Ni foam after the hydrothermal process and annealing at 450 ^°^C. A high density array of MnCo_2_O_4_ nanorods with a length of up to few micrometers was grown on the Ni-foam substrate. Further, it seems that the MnCo_2_O_4_ nanorods are oriented and assembled in a radial form from the center to the surface of micro-spherical superstructure, which looks like chestnut bur-like structures. The spherical structures formed on the surface of the nanorod array have sizes ranging between 1–5 μm. Further, the detailed architecture of the nanorods was observed by TEM as given in Fig. 2c,d. The TEM image clearly shows a chestnut bur-like structure, which is connected to the nanorod array cluster removed from the Ni foam. Further, the selected-area electron diffraction (SAED) pattern of the nanorod array supported chestnut-bur-like structure, given in the inset of Fig. 2c, can be effectively indexed to the spinel polycrystalline structure. The high-resolution transmission electron microscopy (HRTEM) image (Fig. 2d) clearly reveals that the nanorod has a solid architecture without pores and possesses a polycrystalline nature containing planes with different orientations. The calculated interplanar spacings are 0.47 and 0.25 nm for the (111) and (311) planes, respectively, in the spinel MnCo_2_O_4_. Interestingly, the as-obtained chestnut bur-like superstructures cannot be destroyed and broken into discrete individual MnCo_2_O_4_ nanorods by subjecting to long-time ultra-sonication during TEM sample preparation. This suggests that the unique chestnut bur-like superstructures are not a random aggregate of MnCo_2_O_4_ nanorods but the ordered assembly of the nanorods.

**Figure 3:**
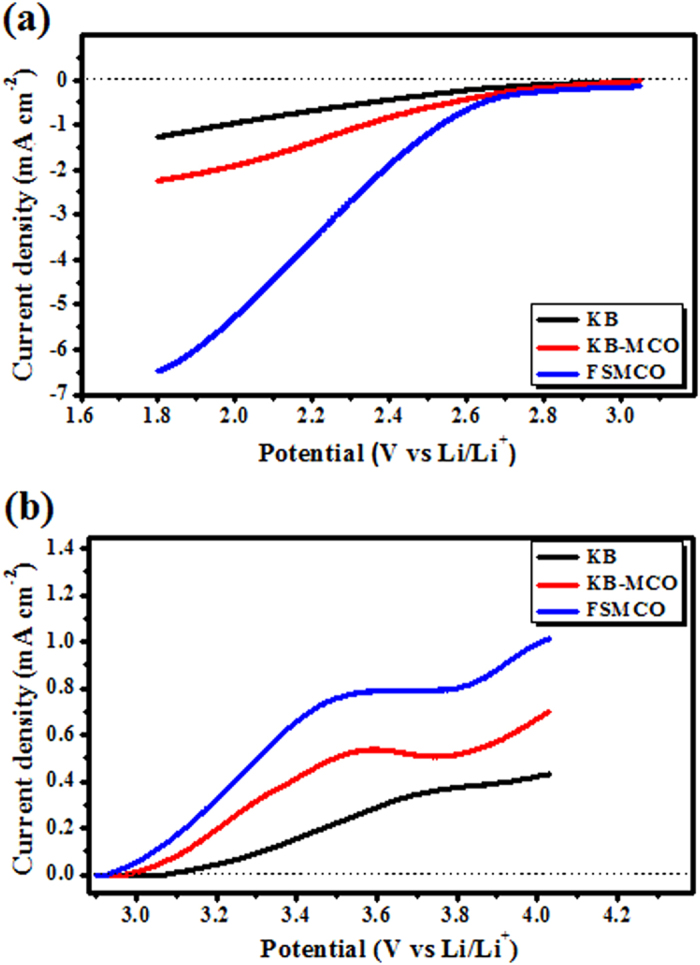
(**a**) Cathodic linear sweep voltammogram and (**b**) anodic linear sweep voltammogram for the free-standing MnCo_2_O_4_ (FSMCO), MnCo_2_O_4_ (MCO)+Ketjen Black (KB), and Ketjen black (KB) electrodes in non-aqueous electrolyte containing 1 M LiTFSI in TEGDME.

The ORR and OER catalytic activities of the free-standing MnCo_2_O_4_ (FSMCO) nanorod arrays formed on Ni foam were assessed in an O_2_-saturated non-aqueous electrolyte of 1 M Bis(trifluoromethane) sulfonimide lithium salt (LiTFSI) in tetra-ethylene glycol dimethyl ether (TEGDME). In addition, the results were compared with the data obtained for the electrode composed of Ketjen black (KB) and the electrode composed of a physical blend of KB and MnCo_2_O_4_ (1:1) (KB-MCO). A commercial three-electrode cell (ECC-air; EL-CELL^®^) using the working electrode of interest, the counter electrode of Li metal and the reference electrode, also of Li metals were used for the tests^23^. Figure 3a shows the cathodic linear sweep voltamograms (LSV) for KB, KB-MCO, and FSMCO measured in 1M LiTFSI in TEGDME at a scan rate of 0.5 mV·s^−1^ in the potential range of 3.1 ~ 1.8 V vs Li/Li^+^. It can be seen that the addition of MnCo_2_O_4_ to the KB electrode resulted in higher ORR onset potential and higher ORR peak current density. Nevertheless, the ORR onset potential and ORR peak current density for the FSMCO electrode were higher than those of the other two electrodes. In particular, the peak current density for the FSMCO electrode (−6.39 mA·cm^−2^) was approximately 3 times higher than that of the KB-MCO electrode (−2.19 mA·cm^−2^) and 5 times higher than that of the KB electrode (−1.25 mA·cm^−2^). Apparently, the free-standing MnCo_2_O_4_ nanorod arrays demonstrated higher activity for ORR compared to KB blended with MnCo_2_O_4_.

The electrocatalysis towards OER for the KB, KB-MCO, and FSMCO electrodes was also investigated as shown in Fig. 3b. Since the OER in a Li-O_2_ battery is closely associated with the decomposition of lithium oxides, the OER activity of KB, KB-MCO, and FSMCO was tested using electrodes on which lithium oxides had been formed through potentiostatic exposure to 2.5 V vs Li/Li^+^ for 1 h. The electrodes were anodically swept from 2.9 to 4.05 V vs Li/Li^+^ at a scan rate of 0.5 mV·s^−1^. In the linear scan for the OER, a small increase in current density with applied potential and a broad peak in the potential range of 3.7 ~ 3.9 vs Li/Li^+^ were observed for the KB electrode. These currents may be associated with the oxygen radical intermediates formed during the oxidation of Li_2_O_2_, which are considered to be responsible for the carbon corrosion^24^. However, broad current peaks with maximum at ~3.54 V were evinced in the potential range of 3.5 ~ 3.6 V vs Li/Li^+^ for FSMCO and KB-MCO electrodes.

In addition, the shift in the peak potential towards lower potential and the increased peak current density were revealed for the FSMCO compared with KB-MCO. Accordingly, the MnCo_2_O_4_ appears to catalyze the oxidation of Li_2_O_2_ effectively and promote the OER process. These results demonstrate that free-standing MnCo_2_O_4_ nanorod arrays have promising ORR/OER electrocatalytic activities in non-aqueous electrolytes. This also confirms that the MCO catalyst can reduce polarization in the Li-O_2_ battery.

**Figure 4:**
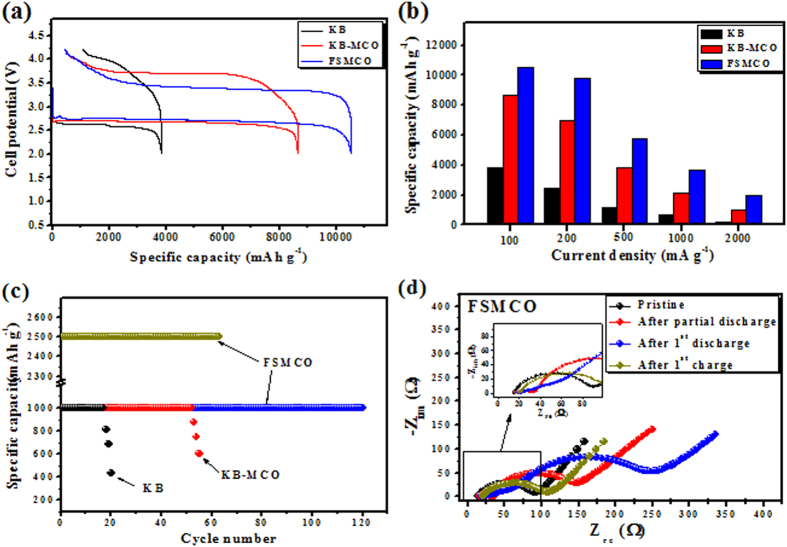
(**a**) 1^st^ discharge-charge curves at current density of 100 mA·g^−1^. (**b**) Discharge capacities as a function of applied current. (**c**) Cyclability measured using the limited capacity discharge mode for the Li-O_2_ cells containing oxygen electrodes composed of only KB, KB-MCO, and FSMCO, respectively. (**d**) Electrochemical impedance spectra for the three-electrode Li-O_2_ cells containing FSMCO oxygen electrodes as a working electrode, obtained after various discharge or charge stages; pristine, after partial discharge up to 1000 mAh g^−1^, after full discharge, and after full charge.

Further, the galvanostatic charge-discharge tests on the Li-O_2_ cell containing the oxygen electrodes composed of KB, KB-MCO, and FSMCO were carried out to evaluate the electrochemical performances of the cells in the potential ranging from 2.0 to 4.2 V. Figure 4a shows the first discharge-charge profiles for the Li-O_2_ cells tested using discharge-charge current density of 100 mA·g^−1^. For the Li-O_2_ cell containing the KB electrode, the first discharge capacity of 3830 mAh·g^−1^ was comparable to that obtained from similar carbon black electrodes in the TEGDME electrolyte^25^,^26^. For the cell containing the KB-MCO electrode, the discharge capacity was improved to 8650 mAh·g^−1^. Furthermore, the discharge capacity of 10520 mAh·g^−1^ corresponding to the specific area capacity 6.8 mAh·cm^−2^ was the highest for the cell containing the FSMCO electrode. In addition, the ORR and OER polarizations in the Li-O_2_ cell were obviously reduced by introducing the MnCo_2_O_4_ (MCO) catalyst to the oxygen electrode, compared with the cell without MCO. The Li–O_2_ cell containing the FSMCO electrode exhibited a discharge potential plateau of 2.79 V, which is higher by 180 and 80 mV than that of the cells containing KB and KB-MCO, respectively. This result clearly indicates that FSMCO can effectively facilitate the ORR and that the catalyst with relatively higher content of MCO is more functional. In the subsequent charge process, the charge potential plateau of the cell with FSMCO was lower by 320 and 720 mV than that of the cells with KB-MCO and KB, respectively. The remarkably decreased overpotentials may be ascribed to the significantly enhanced OER activity on the carbon free FSMCO. In addition, highly porous FSMCO can supply more three-phase (oxygen/catalyst/electrolyte phases, i.e. gas/solid/liquid three-phase) reaction active sites to decompose Li_2_O_2_; especially the chestnut bur-like superstructures, can offer more active sites and enough transmission paths for O_2_ and Li^+^ ions. Although the explicit mechanism for the catalytic behavior has not yet been elucidated^27^, we ascertain that MCO can accelerate the kinetics of both the ORR and OER, which should be ascribed to this unique combination and hierarchical structure of MnCo_2_O_4_.

The rate capability of the Li-O_2_ cells containing three different oxygen electrodes was examined, and the results are shown in Fig. 4b. It can be demonstrated that the capacity for FSMCO was almost retained even using double current density, suggesting that O_2_ diffusion and Li_2_O_2_ accommodation was efficient in the hierarchical porous structure. Nevertheless, the capacity significantly decreased to 2015 mAh·g^−1^ when a higher current density of 2000 mA·g^−1^ was applied. In contrast, the discharge capacity was generally much smaller for KB-MCO and KB than for FSMCO. The plateau cell potentials measured during discharge and charge for the Li-O_2_ cells containing KB-MCO and FSMCO electrodes, respectively, are shown in Supplementary Fig. S3. Interestingly, FSMCO displayed superior ORR activities, not only in specific capacities but also in plateau potentials compared to KB-MCO catalysts. Moreover, the charge-discharge potential gap of about 0.65 V observed at 100 mA·g^−1^ was comparable to that reported for precious metal based catalysts such as Au/C, Pt/C^27^, Au/Pt nanoparticles^28^,^29^ and Ru-graphene^30^, and lower than that for other reported catalysts such as carbonous materials^31^,^32^,^33^, metal oxides and their carbonous composites^13^,^14^,^15^,^16^,^17^,^18^,^19^,^20^,^21^,^34^,^35^, pyrochlore^36^, MnCo_2_O_4_-graphene^22^ and mesoporous MnCo_2_O_4_^37^ at a comparable applied current in similar ether based electrolytes.

Long-term cyclability is another requirement for secondary Li–O_2_ batteries. However, it is not easy to achieve this goal due to the inevitable formation of by-products, the instability of the electrolytes, and the incomplete decomposition of lithium oxides^25^,^38^. Supplementary Fig. S4 shows the potential profiles recorded during cyclability tests of Li-O_2_ cells containing KB-MCO and FSMCO tested in fully discharge mode. The cell with FSMCO could exhibit good reversibility up to the 10^th^ cycle while maintaining a capacity of over 4000 mAh·g^−1^, while fast capacity fading was observed for the cell with KB-MCO. In addition, the capacity degradation observed for the cell with FSMCO after 10^th^ cycle was mainly attributed to the instability of the TEGDME at high potential^39^. It is already proved that the solvent used to prepare electrolyte in present study, i.e TEGDME, is also not stable in the operating potential window of a Li–O_2_ battery and is consumed during the charging process of a Li–O_2_ cell even at moderate potentials^40^. The better cycle life achieved for cell with FSMCO, when tested at full charge–discharge mode, is ascribed to the reduced parasitic reactions between Li_2_O_2_ and carbon to cause the formation of resistive carbonate at the interface and the decomposition of binder material by oxygen radical intermediates to LiOH and LiF, responsible for poor cycleability. The enhanced electrochemical properties including cyclability of the cell with FSMCO are also attributed to the unique open framework of the FSMCO nanorod arrays, which allows the oxygen to easily access the inner space of the electrode. Moreover, the hierarchical porous structure can prevent clogging caused by the discharge products and promote the surface reaction of the formation and decomposition of Li_2_O_2_ on the FSMCO. Figure 4c shows the cyclability of Li-O_2_ cells containing pure KB, KB-MCO, and FSMCO electrodes tested at the applied specific current of 500 mA·g^−1^ under a limited discharge-charge capacity mode of 1000 mAh·g^−1^. The cycle life of the Li-O_2_ cell was extended to 119 cycles when the binder-free and carbon-free spinel MnCo_2_O_4_ oxide, i.e. FSMCO, was employed in the oxygen electrode. The Li-O_2_ cell containing the KB-MCO electrode exhibited a cycle life longer than 55 cycles with the capacity of 1000 mAh·g^−1^. In contrast, the capacity of the Li-O_2_ cell containing the KB electrode was maintained for only 17 cycles. The charge-discharge potential profiles with the cycle for the Li-O_2_ cells with KB, KB-MCO, and FSMCO are presented in Supplementary Fig. S5. For the cell containing FSMCO, up to 100 cycles, the end potential in each discharge segment was stabilized to 2.6–2.7 V, and the end potential in each charge segment was in the range of 3.9–4 V (Supplementary Fig. S5c). In contrast, for the cell with KB-MCO, the discharge end potential of each cycle was stabilized to 2.5–2.6 V, and the end potential of each charge segment was in the range of 4–4.2 V (Supplementary Fig. S5b). Further, the battery testing was extended for Li-O_2_ cell containing FSMCO electrode under the capacity limit of 2500 mAh·g^−1^ to determine the cycling response. Figure 4c and Supplementary Fig. S5d present the result of test runs under the capacity limit of 2500 mAh·g^−1^, demonstrating 63 stable cycles, supporting the good cycle life of Li-O_2_ cells and confirming the highly reversible formation and decomposition of Li_2_O_2_ on the FSMCO electrode. The above results reveal that the Li-O_2_ cell can operate reversibly with fast kinetics, assuring performances hardly seen in earlier reports on Li-air batteries containing non-precious catalysts. It is believed that this exceptional behavior originates from the integrated design of the advanced oxygen electrode. The good cycling performance of the FSMCO cell can also be attributed to the direct connection between the MnCo_2_O_4_ nanorods and the Ni-substrate, which can greatly increase the electron transport in the electrocatalyst compared with the electrodes, where catalysts are mixed with carbon. The terminal discharge potential even after 119 cycles was still higher than 2.0 V, the cut-off potential used in the full discharge mode. This terminal discharge potential for the FSMCO cell was much higher than that for the KB cell maintained only for 17 cycles, revealing that FSMCO is an excellent catalyst compared with earlier reported porous MnCo_2_O_4_ microsphere and MnCo_2_O_4_-graphene hybrid catalysts^22^,^37^.

Electrochemical impedance spectroscopy (EIS) was employed to monitor the formation and decomposition of Li_2_O_2_ in the oxygen electrode of the Li-O_2_ cells. The electrochemical impedance spectra obtained for the three-electrode Li-O_2_ cells containing FSMCO and KB-MCO electrodes in four different charge-discharge process stages are shown in Fig. 4d and Supplementary Fig. S6, respectively. The equivalent electric circuit^41^ used to model the EIS curves is also shown in Supplementary Fig. S7. For both the FSMCO (Fig. 4d) and KB-MCO electrodes (Supplementary Fig. S6), it can be seen that all the EIS curves after discharge-charge process are composed of two consecutive semicircles in the high frequency and medium frequency region. The semicircle observed in high frequency region appears to be associated with film formation including mainly accumulation of discharge products such as Li_2_O_2_ and formation of solid electrolyte interphase (SEI) layer by the electrolyte decomposition on the electrode^42^. The intercept of this semicircle on the real axis mainly represents the electrolyte resistance (R_e_) and the diameter of the semicircle is related to the interfacial resistance (R_1_) related to the covering of Li_2_O_2_ and SEI layers. The medium frequency semicircle is related to the time constant of charge-transfer resistance and double-layer capacitance on the composite electrode/electrolyte surface. The diameter of the medium frequency semicircle represents the charge-transfer resistance for oxygen reduction reaction (R_2_)^41^. At low frequencies, the asymptotic limit of the real part of the impedance is the total impedance (R_t_) in the overall process, i.e. the intercept of semicircle at lower frequency. The medium frequency semicircle is followed by a sloped line in low frequency range is due to Z_w_, which is characteristic of a diffusion controlled process due to the diffusion of lithium ions and also gaseous oxygen within the porous oxygen electrode described by a finite Warburg element Z_w_^42^. The values of the resistive components in the impedance spectra were computed by fitting the model to the experimental data and the obtained parameters were tabulated in Supplementary Table S1. On analysis of each component in impedance plot (Supplementary Table S1), for both the cells, the electrolyte resistance (R_e_) did not change significantly after the entire discharge-charge process, while the interfacial resistance R_1_ increased with the depth of discharge. This is due to the formation of resistive discharge product and accumulation in the pore structure of the oxygen electrode^42^. Similarly, the charge transfer resistance (R_2_) significantly increased with discharge from 64.8 to 276.1 Ω cm^−2^ for KB-MCO and from 87.7 to 210 Ω cm^−2^ for FSMCO, respectively. The high charge-transfer resistance for both the electrodes is attributed to the accumulation of highly resistive reaction product on the surface^43^. However, after charging, the high and medium frequency semicircles on impedance spectra revealed significant decrease in R_1_ and R_2_, due to the decomposition of discharge product.

In addition, the electrochemical impedance spectra of a pristine electrode and the electrodes after the first charge were quite similar, while the impedance of the electrode after the first discharge increased significantly compared to that of the pristine electrodes. The increase in the total impedance (R_t_) after discharging is as expected and in agreement with the literature^41^, which is due to the formation of resistive product Li_2_O_2_^44^. Nevertheless, as seen from the impedance plot for the electrode after charging, the Li_2_O_2_ formed during discharge can be considerably decomposed during the charging process. Furthermore, from the plot (Supplementary Fig. S8), the variation in the R_t_ for KB-MCO after cycling was much higher than that for FSMCO. This larger increase in the R_t_ may be attributed to the corrosion of Ketjen black to form Li_2_CO_3_ in addition to the electrolyte decomposition^38^,^45^,^46^.

## Discussion

**Figure 5:**
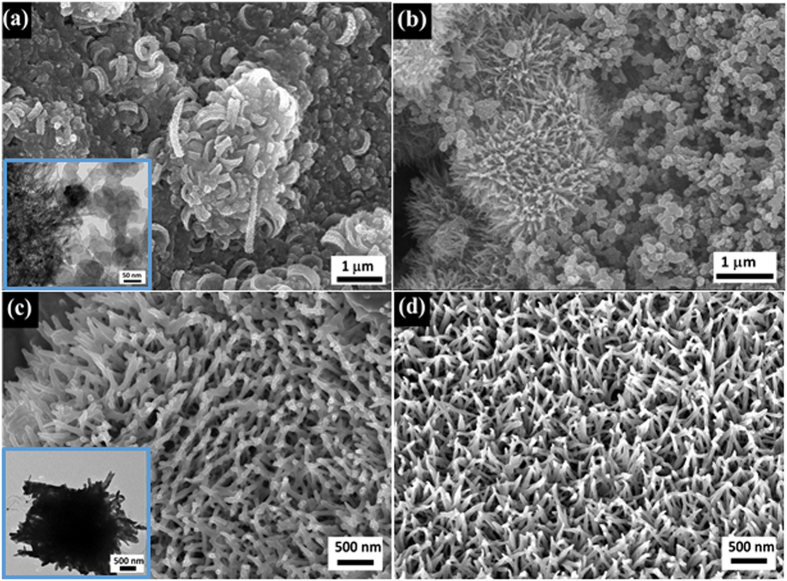
FE-SEM images of the surface of the KB-MCO electrode. (**a**) after the 1^st^ full discharge; and (**b**) charge, and the FSMCO electrode; (**c**) after the discharge; and (**d**) charge. Insets in (**a**,**c**) show corresponding TEM images.

FE-SEM and TEM images were obtained to investigate the change in surface of the KB-MCO and FSMCO electrodes after the 1^st^ full discharge and charge cycle. Figure 5 shows the FE-SEM and TEM images observed for the KB-MCO and FSMCO electrodes after full discharge and charge. On the whole, these two electrodes exhibited quite different morphologies. For the FSMCO electrode, the nanorods appear to be covered evenly with discharge products (Fig. 5c), whereas distinct toroid shaped discharge products were formed on the KB-MCO electrode. Specifically, the toroid shape was found to consist of nano-flakes. Toroid-like morphologies of Li_2_O_2_ have also been reported for the electrode containing KB as the carbon source^36^,^47^. Further, the nature of the discharge products formed on the surface of the electrodes was analyzed using XRD. The crystalline character of the discharge product was confirmed for the KB-MCO electrode. The peaks obtained in the pattern corresponded to the (100) and (101) planes of crystalline Li_2_O_2_ as shown in Supplementary Fig. S9. However, for the FSMCO, no additional peak was observed in the XRD pattern (Supplementary Fig. S9), which indicates the low degree of crystallinity of the discharge product. Furthermore, the formation of Li_2_O_2_ was verified by Raman spectroscopy. The presence of a peak at 780 cm^−1^ in the Raman plot (Supplementary Fig. S10) and the absence of a peak after charging confirms the reversibility of the amorphous Li_2_O_2_ formation on the FSMCO surface. As confirmed in Fig. 5b,d, the Li_2_O_2_ disappeared when the cell was charged to 4.2 V and, in particular, the bare MnCo_2_O_4_ nanorods reappeared for FSMCO. After charging, the formed discharge product is oxidized and the surface becomes ready for the next ORR process. These results imply that the FS-MCO catalyst without carbon affects both the morphology and crystallinity of the discharge product of Li_2_O_2_ simultaneously, thus improving the reversibility in each cycle through increased contact area between Li_2_O_2_ and the catalyst.

**Figure 6:**
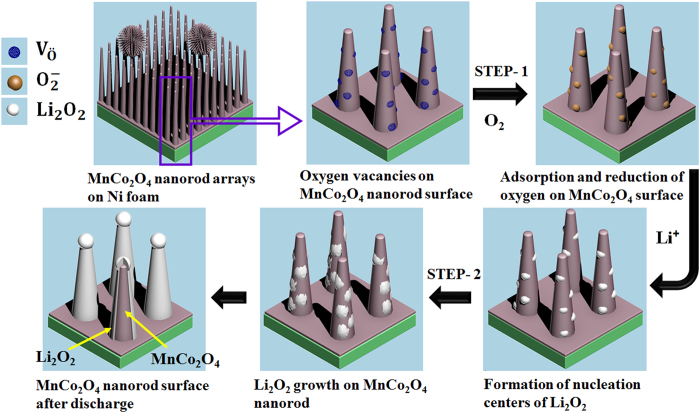
Schematic illustration of the nucleation and growth of Li_2_O_2_ on the MnCo_2_O_4_ nanorod array.

Interestingly, the morphological evolution of the discharge product on the FSMCO electrode was highly reversible during the discharge–charge processes. The morphological evolution and the possible growth mechanism of Li_2_O_2_ on the MnCo_2_O_4_ nanorods can be explained as shown in the schematic illustration of Fig. 6. The growth process of Li_2_O_2_ can be simply divided into two steps. In an initial step, the nucleation sites are formed on the surface of MnCo_2_O_4_ nanorods and in the subsequent step, Li_2_O_2_ grows laterally to completely cover the nanorods. As suggested previously, oxygen diffuses through the oxygen electrodes is bound to the surface of the nanorods via absorption at oxygen vacancies formed due to solid-state redox couples of Mn^2+^/Mn^3+^and Co^3+^/Co^2+^, and then reduced to form meta-stable O_2_^−^ in the discharge process^35^,^36^. Finally, Li_2_O_2_ is generated by the subsequent dismutase reaction of superoxide ions with solvated lithium ions in the liquid electrolyte. The extended growth of the formed nucleation site in the preceding step results in the complete coverage of nanorods with the discharge product, as shown in Fig. 5c. Moreover, it can be seen that the discharge products formed on the top of the nanorods are larger than those formed at any other place. This indicates that the open ends of the nanorods are the most active sites due to a higher surface-charge density. Similarly, the most electrochemically active sites at the tips have been reported for NiCo_2_O_4_, resulting in the formation of Li_2_O_2_ microspheres with nanoflake-like morphology^21^.

Furthermore, to obtain insight into the cause of the failure of the Li-O_2_ cells, the Li-metal and FSMCO electrode were examined after disassembling the cell in a glove box. Supplementary Fig. S11a shows photographs and FE-SEM images of the FSMCO electrode after 119 cycles. They clearly show the porous structure of the FSMCO electrode (Inset of Supplementary Fig. S11a). Moreover, the cycling of the reassembled Li-O_2_ cell with fresh Li-metal and electrolyte for more than 30 cycles under limited capacity mode (Supplementary Fig. S11b) indicates that the failure of the Li-O_2_ cell was not due to the oxygen electrode, and might be due to the corrosion of the Li anode. Hence, the Li-anode after cell fade was examined using XRD and FE-SEM. The degradation of the Li-metal due to its corrosion is clearly shown in Supplementary Fig. S12. As evinced in the inset image (Supplementary Fig. S12b), the Li-metal was covered with a white layer of LiOH and Li_2_CO_3_, as confirmed by XRD and Raman spectroscopy (Supplementary Fig. S12c and d). The corrosion of the Li anode due to the oxygen crossover effect and formation of LiOH and Li_2_CO_3_ has been reported in previous work^48^. In addition, the gradual decomposition of TEGDME by the ORR intermediate (i.e., consumption of electrolyte) may be one of the reasons for the failure of the cell^46^. Hence, a stable electrolyte in combination with the carbon and binder free electrode can further improve the cyclability of Li-O_2_ cells. The results presented herein demonstrate a special example of the evolution characteristics of Li_2_O_2_ and provide a clear visualization of the electrochemically active sites for the oxygen electrode.

In summary, hierarchical nanorods of MnCo_2_O_4_ with a chestnut bur-like structure, as an oxygen electrode catalyst, grown directly on Ni foam have overcome the challenge of OER in a non-aqueous Li–O_2_ battery and contributed to make a high-performance oxygen electrode with a reversible capacity of up to 10520 mAh·g^−1^. The enhanced catalytic activity of the free-standing MnCo_2_O_4_ facilitated more efficient decomposition of the discharge reaction products of Li_2_O_2_, which was reflected in the low charging overpotentials, the resultant increased round-trip efficiency, and the improved cycle life of more than 115 cycles. The high reversibility and large capacity of the free-standing MnCo_2_O_4_ based Li-O_2_ cell is a direct outcome of its novel features which are crucial for catalytic reactions: good conductivity and the existence of porosity that enables good diffusion of the oxygen and electrolyte to the active sites. The present investigation is important as it demonstrates that the performance of the Li-O_2_ batteries can be thoroughly recovered by engineering oxygen electrode design and further improvements can also be achieved by renewing the electrolyte; this offers a new pathway to understand the key elementary reactions in Li-O_2_ batteries.

## Methods

### Synthesis of free-standing electrode

The binder- and carbon-free electrodes were synthesized using a simple urea assisted hydrothermal method. The precursor solution was prepared by dissolving 1 mM MnSO_4_.6H_2_O and 2.5 mM (CH_3_COO)_2_Co in a 50 ml mixture of ethyl alcohol and deionized (DI) water with a volume ratio of 1:4. Urea was then added to the resultant solution while stirring and maintaining the ratio of reaction promoter to metal ions at 10. After stirring for 1 h, the obtained homogeneous solution was transferred to a Teflon lined stainless steel autoclave with the capacity of 70 ml. To grow free-standing ternary spinel MnCo_2_O_4_ on Ni foam, pieces of Ni foam were placed vertically in the solution and the entire assembly was maintained at 140 ^°^C for 10 h. After completion of the hydrothermal reaction, the foamed Ni electrodes covered with MnCo_2_O_4_ precipitates were collected from the autoclave and rinsed with deionized (DI) water to remove ionic species before drying in a vacuum oven overnight. Finally, the obtained electrodes were annealed in air to form the desired phase. The loading weight of MnCo_2_O_4_ is calculated by subtracting the weight of bare Ni-foam subjected to the similar annealing condition from that of MnCo_2_O_4_ coated Ni foam.

### Material characterization

Thermo-gravimetric analysis (TGA) was carried out using a thermogravimetric analyzer (Perkin Elmer TGA7) at a scan rate of 10 ^°^C·min^−1^ in air atmosphere. The crystal structure of the annealed samples was characterized by X-ray diffraction (XRD, Rigaku DIII Ultima with Cu Kα radiation) with 2θ ranging from 10^°^–80^°^. Further, the microstructure of the as prepared free standing electrode and the electrodes after tests was observed using field-emission scanning electron microscopy (FE-SEM, S-4700 Hitachi) equipped with energy dispersive spectroscopy (EDS) and high–resolution transmission electron microscopy (HR-TEM, Philips Tecnai F20 at 200 kV). Raman analysis was also carried out using a Raman spectroscopy (Horiba Jobin Yvon HR800 with 744 nm initial excitation laser) (KBSI Gwangju-center).

### Electrochemical analysis

The Li-O_2_ cells in the form of CR-2032 coin cells were fabricated in an Ar-filled glove box. Tetra ethylene glycol dimethyl ether (TEGDME) containing 1M lithium bis (trifloromethane) sulfonamide (LiTFSI) was used as the electrolyte. A glass microfiber separator (Whatman, GF/F, 150) soaked with the electrolyte was placed between the lithium metal and the free-standing MnCo_2_O_4_ oxygen electrode with average loading of 0.8 ~ 1 mg. Galvanostatic charge-discharge tests were carried out using a battery cycler (Won-A-Tech WBCS 3000) under a current density of 100 ~ 2000 mA·g^−1^. The impedance analysis of the Li-O_2_ cells was conducted before and after charge-discharge cycling using an impedance analyzer (ZIVE SP2 instrument; Won-A-Tech) in the frequency range of 1 Hz ~ 1 MHz with an amplitude of 10 mV. For measuring the impedance of Li-O2 cell at various condition during discharge-charge process.

The oxygen reduction reaction (ORR) and oxygen evolution reaction (OER) activity of the free-standing MnCo_2_O_4_ electrode in the LiTFSi-TEGDME electrolyte was analyzed through linear voltammetry tests at the scan rate of 0.5 mV·s^−1^ using a commercial three-electrode Li-O_2_ cell (ECC-Air; EL-CELL^®^), where two Li metals were used as the counter electrode and the reference electrode, respectively, and the free-standing MnCo_2_O_4_ oxygen electrode was used as the working electrode of interest. Moreover, this three electrode Li-O_2_ cell was used to record electrochemical impedance spectra after various discharge-charge stages.

To analyze the reaction products formed on the oxygen electrode, the cell was disassembled after testing, and the collected oxygen electrode was rinsed with solvent to remove salts before characterization. The microstructure and morphology of the reaction products were analyzed using SEM and XRD. Raman analysis was carried out to determine the phase of the discharge product.

## Additional Information

**How to cite this article**: Kalubarme, R. S. *et al.* Simple synthesis of highly catalytic carbon-free MnCo_2_O_4_@Ni as an oxygen electrode for rechargeable Li-O_2_ batteries with long-term stability. *Sci. Rep.* **5**, 13266; doi: 10.1038/srep13266 (2015).

## Supplementary Material

Supplementary Information
