# Supplementary material for: Simple synthesis of highly catalytic carbon-free MnCo2O4@Ni as an oxygen electrode for rechargeable Li–O2 batteries with long-term stability
Source: Sci Rep. 2015 Aug 21;5:13266. doi: 10.1038/srep13266 (PMC4543937; doi:10.1038/srep13266)
Supplement: Supplementary Information [file srep13266-s1.pdf]

Supporting Information for

Simpe synthesis of highly catalytic $MnCo_2O_4$@Ni as an oxygen electrode for rechargeable Li–O_2 Batteries with long-term stability

Ramchandra S. Kalubarme [a], *Harsharaj S. Jadhav* [a], *Duc Tung Ngo* [a], *Ga-Eun Park* [a], *John G. Fisher* [a], *Yun-Il Choi* [b], *Won-Hee Ryu* [c], *and Chan-Jin Park* [a,*]

[a] Department of Material Science and Engineering, Chonnam National University, 77, Yongbongro Bukgu Gwangju 500-757, South Korea

[b] Central Research Center, Doosan Heavy Industries & Construction Co., 22, Doosan volvoro, Seongsangu, Changwon 642-792, Gyeongsangnamdo, South Korea

[c] Department of Chemical and Environmental Engineering, Yale University, New Haven, Connecticut 06520-8286, United States

Supplementary Figure S1

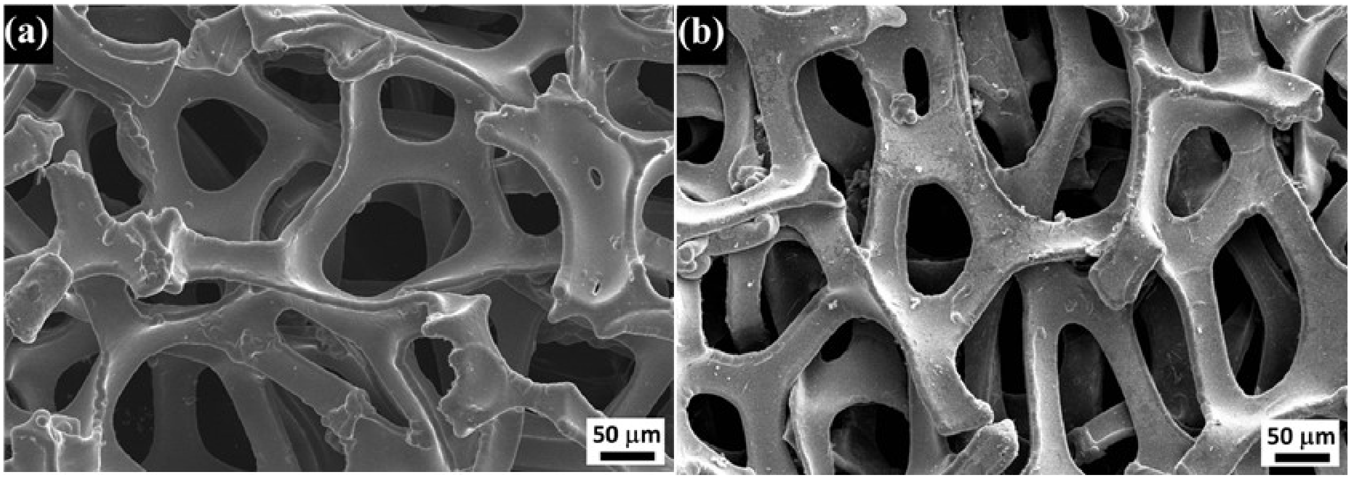

Fig. S1. SEM images of the surface of (a) the pristine etched Ni foam and (b) the Ni foam after immersion in precursor solution at 140 ^{o}C for 1 h.

Supplementary Figure S2

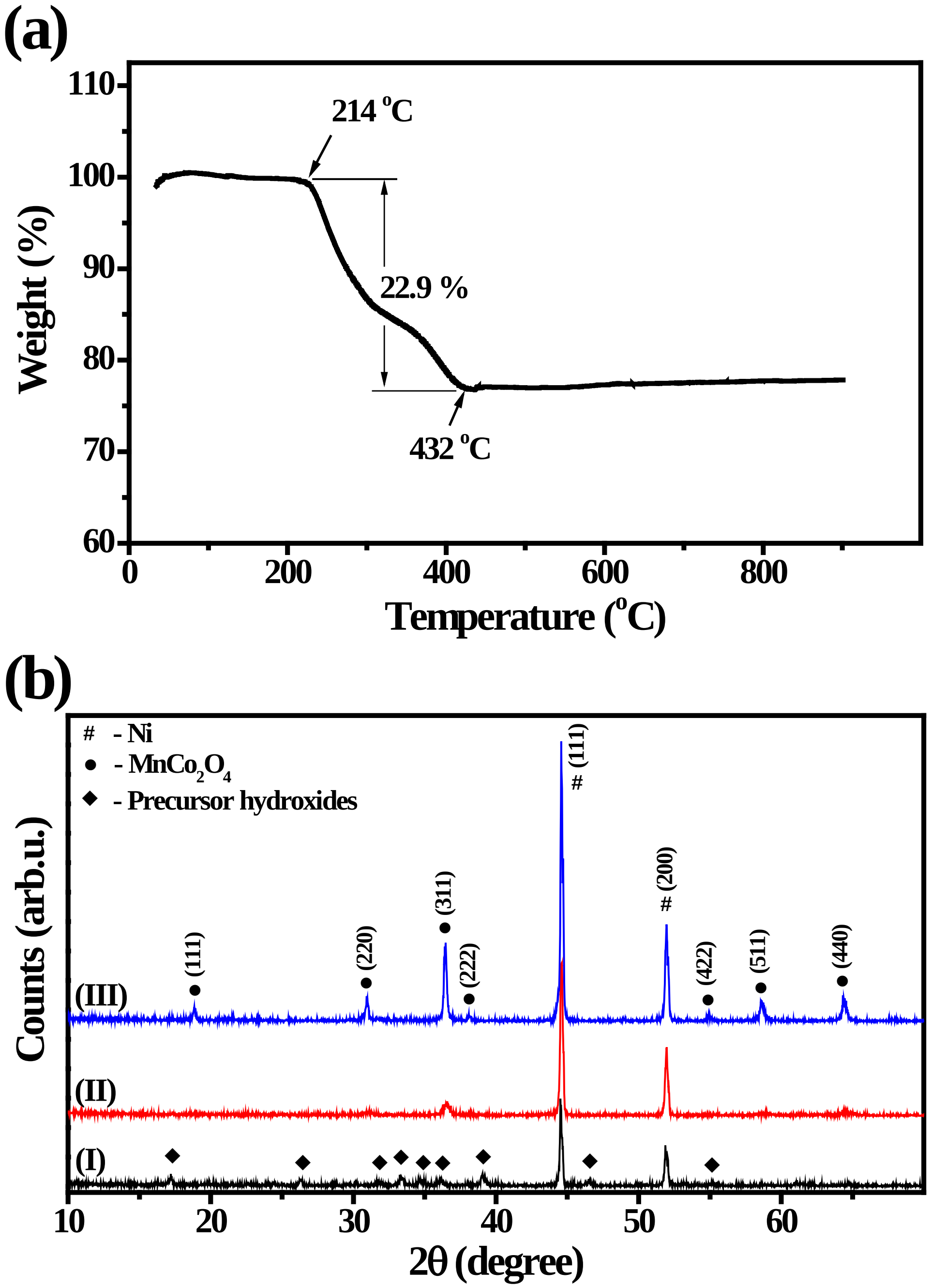

Fig. S2. (a) Thermo-gravimetric analysis of the hydroxide powders obtained via hydrothermal synthesis, measured between 30-900 °C using a temperature sweep rate of 10 $^{o}C \cdot min^{-1}$; (b) X-ray diffraction patterns of the nanorod arrays formed on Ni foam; (I) as prepared, after annealing at (II) 425 °C and (III) 450 °C, respectively.

Supplementary Figure S3

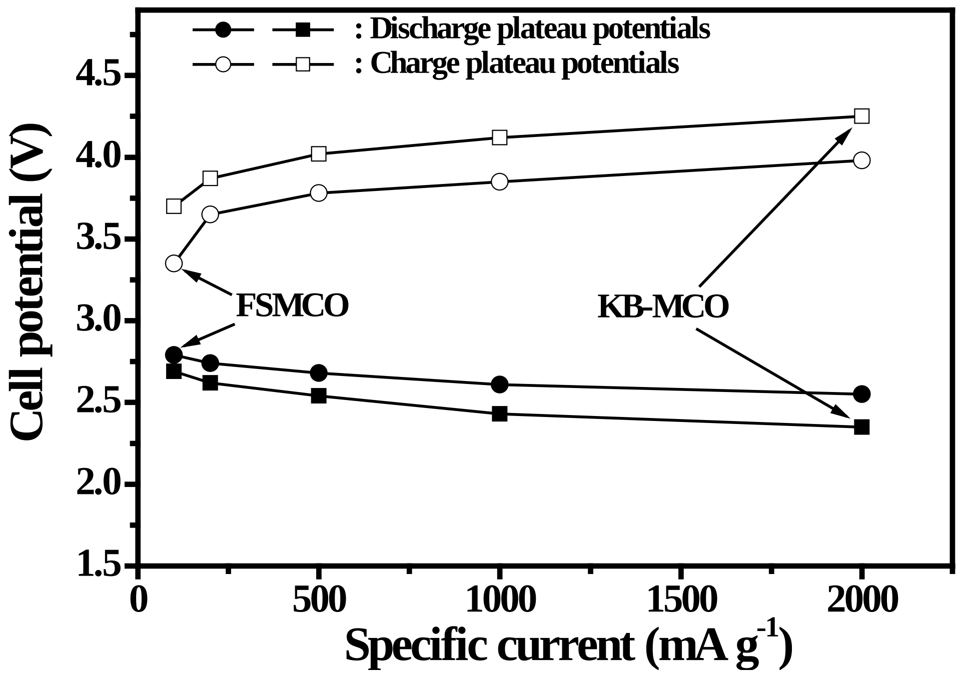

Fig. S3. Plateau cell potentials measured during discharge and charge for the Li-O_2 cells containing KB-MCO and FSMCO electrodes, respectively, as a function of applied specific current.

Supplementary Figure S4

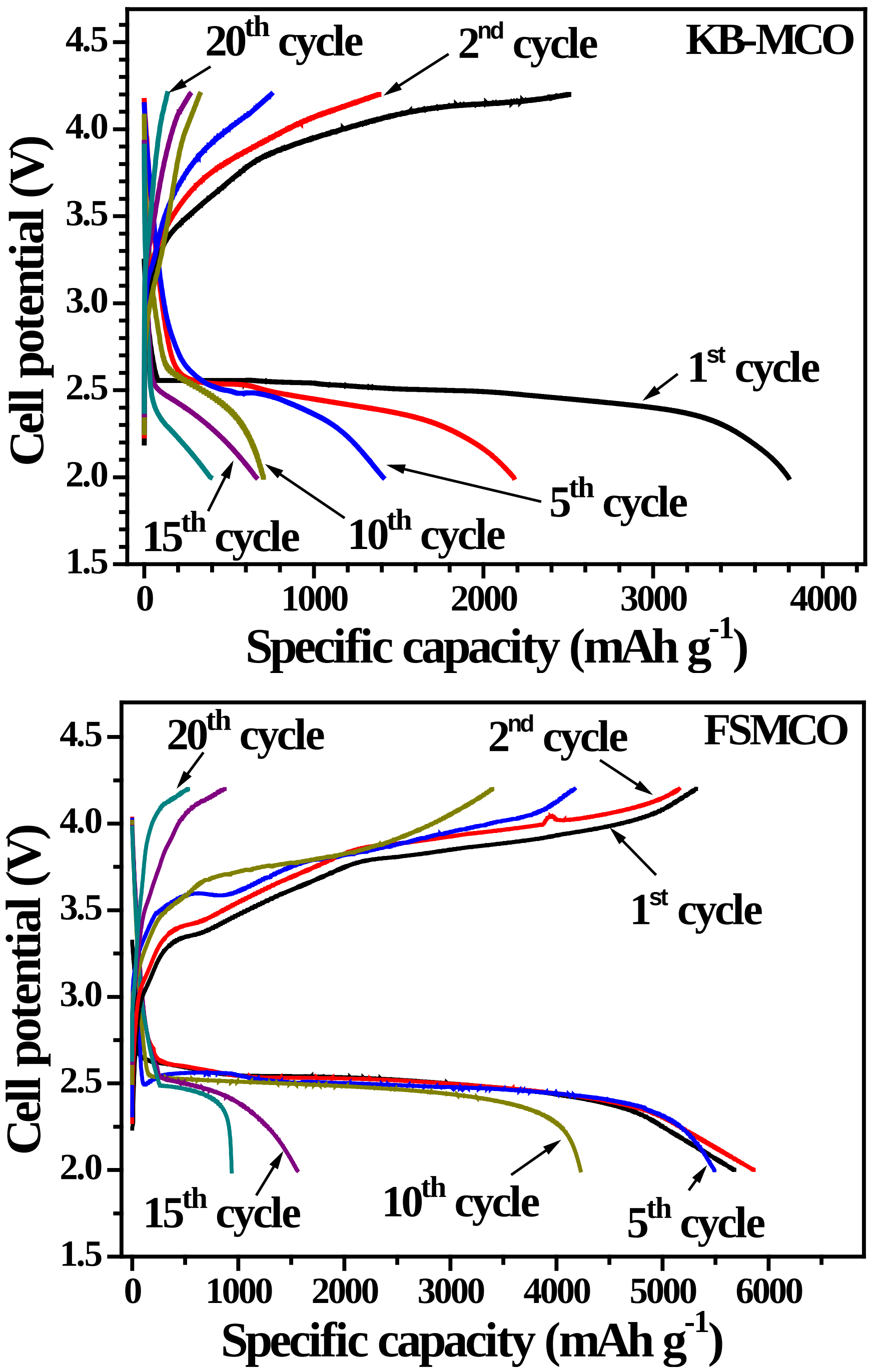

Fig. S4. Cyclability of Li-O_2 cells containing KB-MCO and FSMCO tested using full discharge mode at applied current of 500 mA cm^{-2}

Supplementary Figure S5

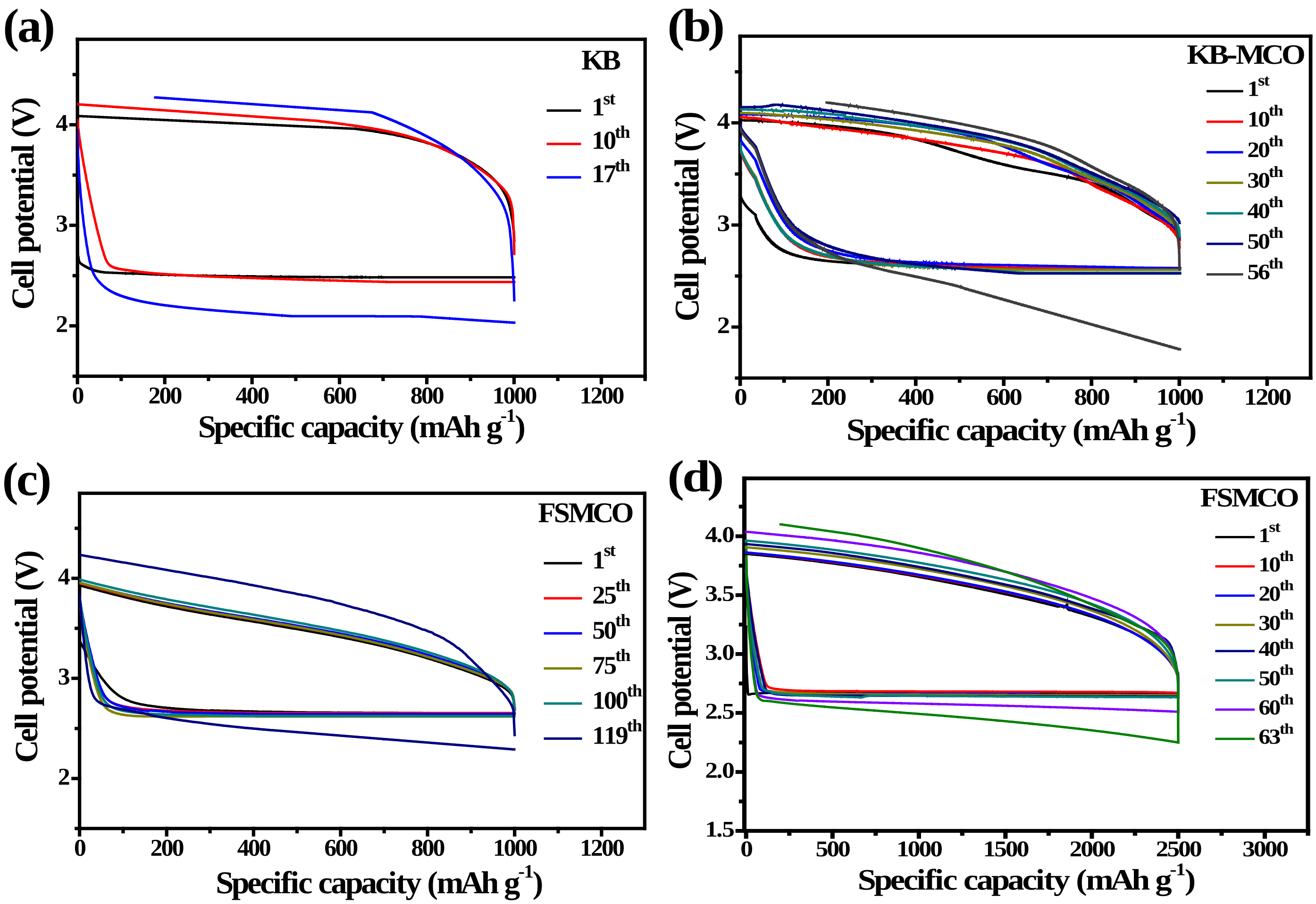

Fig. S5. Charge-discharge potential profiles with the cycles for the Li-O_2 cells with (a) KB and (b) KB-MCO oxygen electrode tested at an applied specific current of 500 mA·g^{-1} using the limited capacity mode of 1000 mAh·g^{-1}, and FSMCO oxygen electrode tested at current density of 500 mA·g^{-1} using the limited capacity mode of (c) 1000 and (d) 2500 mAh·g^{-1}.

Supplementary Figure S6

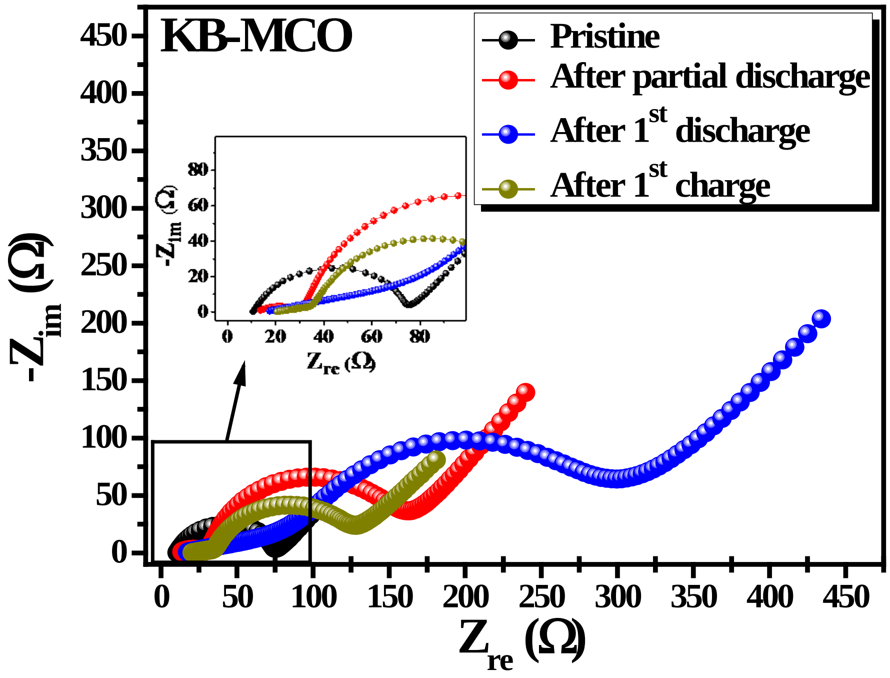

Fig. S6. Electrochemical impedance spectra for the three-electrode Li-O_2 cells containing KB-MCO oxygen electrodes as a working electrode, obtained after various discharge or charge stages; pristine, after partial discharge up to 1000 mAh g^{-1}, after full discharge, and after full charge.

Supplementary Figure S7

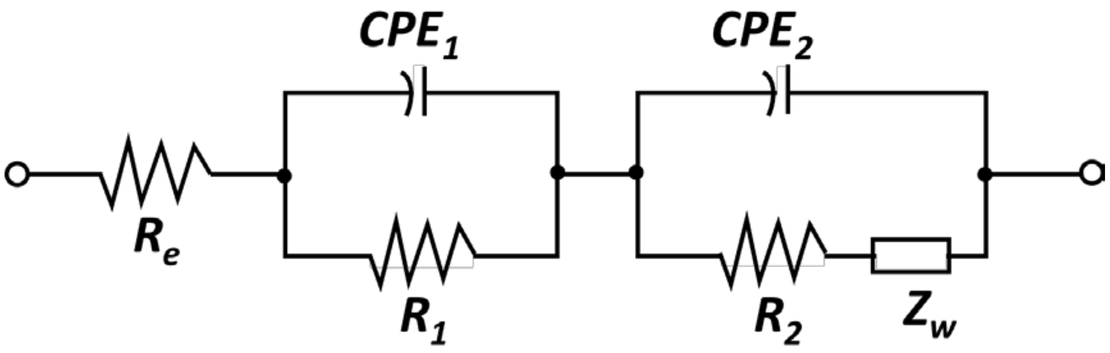

Fig. S7. Equivalent circuit used to model the impedance spectra.

In the circuit, R_e represents mainly the electrolyte resistance. The elements R_1 and R_2 represent the interfacial resistance related to the covering of Li_2O_2 and SEI layers and the charge transfer resistance for ORR, respectively. CPE_1 and CPE_2 are the corresponding constant phase elements. Z_w is the Warburg element reflecting diffusion controlled process for Li-ion and gaseous oxygen.

Supplementary Table S1

Table S1. Electrolyte resistance (R_e), interfacial resistance (R_1), and charge transfer resistance (R_2) calculated from impedance plots by fitting using the equivalent circuit.

Sample	State	R_e (Ω cm^{-2})	R_1 (Ω cm^{-2})	R_2 (Ω cm^{-2})
**FSMCO**	Pristine	13.6	1.9	87.7
	Partially discharged	14.3	26.8	113
	Discharged	15.8	111.1	210
	Charged	17.4	6.5	99.4
**KB-MCO**	Pristine	9.6	1.1	64.8
	Partially discharged	11.7	33.5	145
	Discharged	13.2	92.8	276.1
	Charged	15.1	21.15	121.5

Supplementary Figure S8

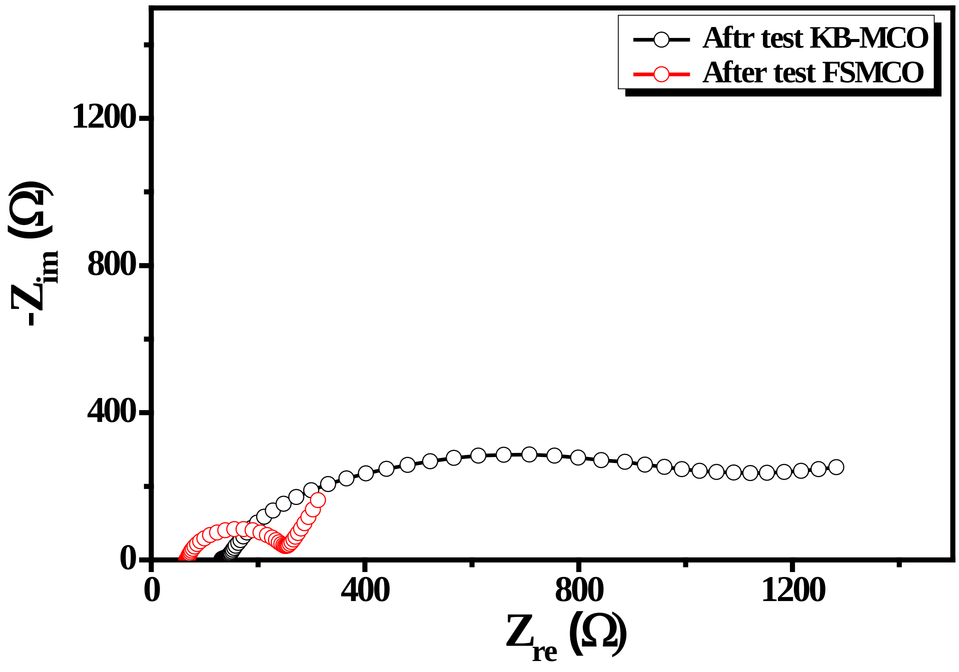

Fig. S8. Electrochemical impedance spectra for the Li-O_2 cells containing KB-MCO and FSMCO after testing.

Supplementary Figure S9

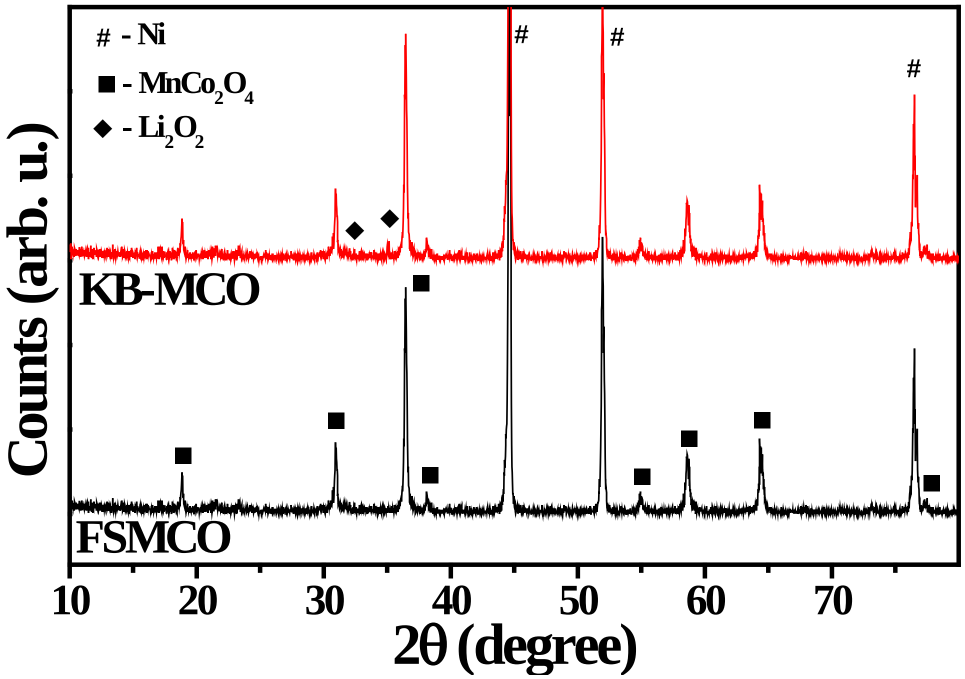

Fig. S9. XRD patterns of the discharged KB-MCO and FSMCO electrodes.

Supplementary Figure S10

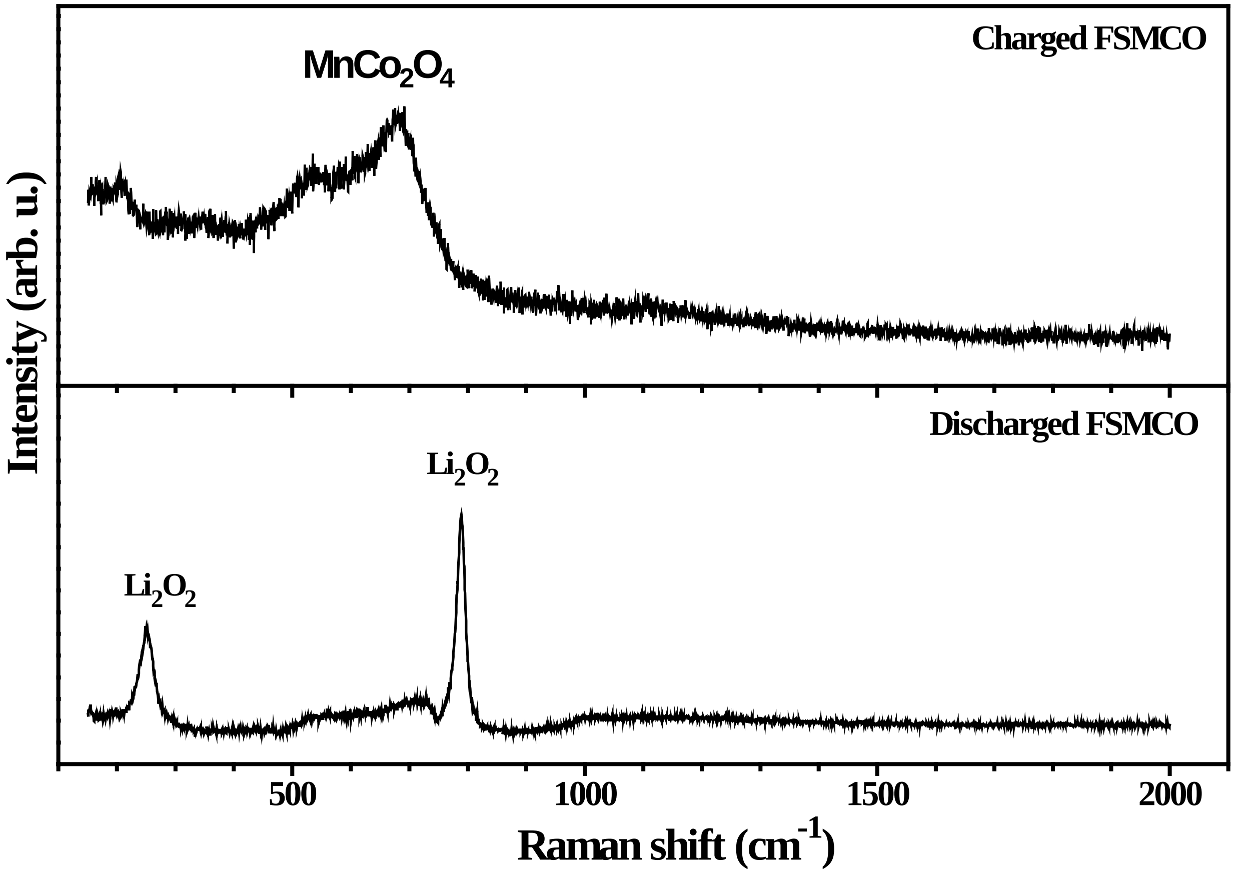

Fig. S10. Raman spectra of the FSMCO electrode in discharged and charged states.

Supplementary Figure S11

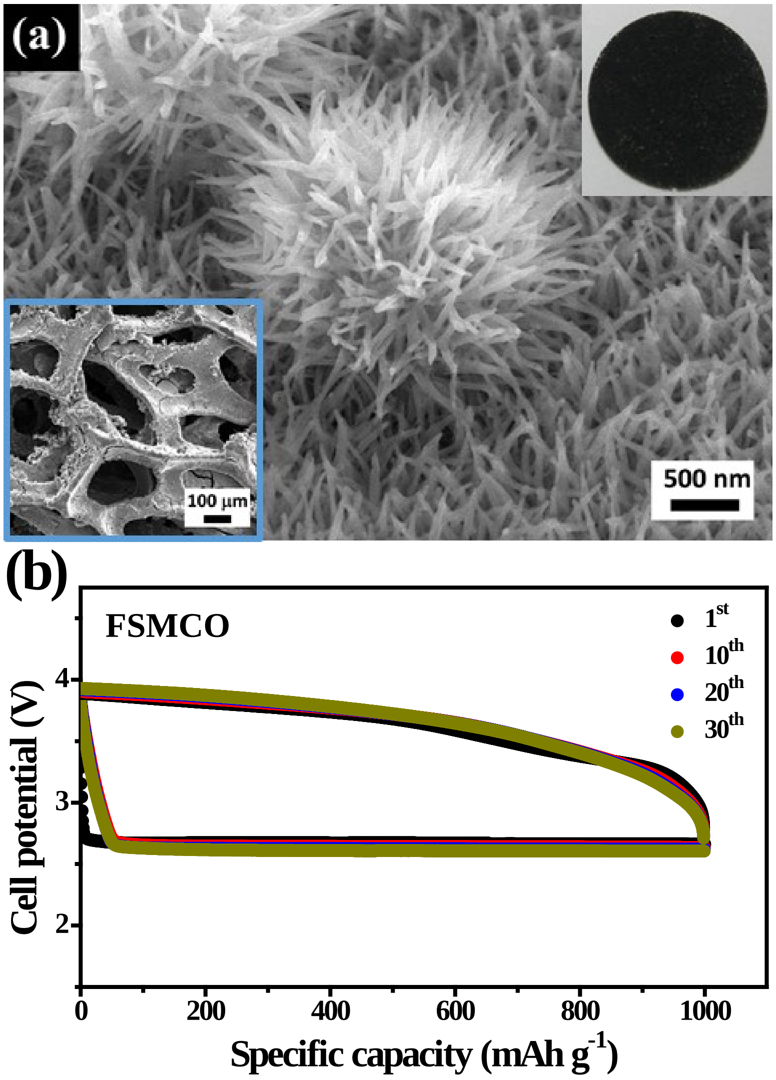

Fig. S11. (a) FE-SEM image of FSMCO oxygen electrode after cell fade (Inset: At top right corner- photograph and at bottom left corner- low magnification image) and (b) Potential profile for reassembled Li-O_2 cell consisting of a used FSMCO electrode (119 cycles old) and new Li-metal, and an electrolyte tested in constant capacity mode.

Supplementary Figure S12

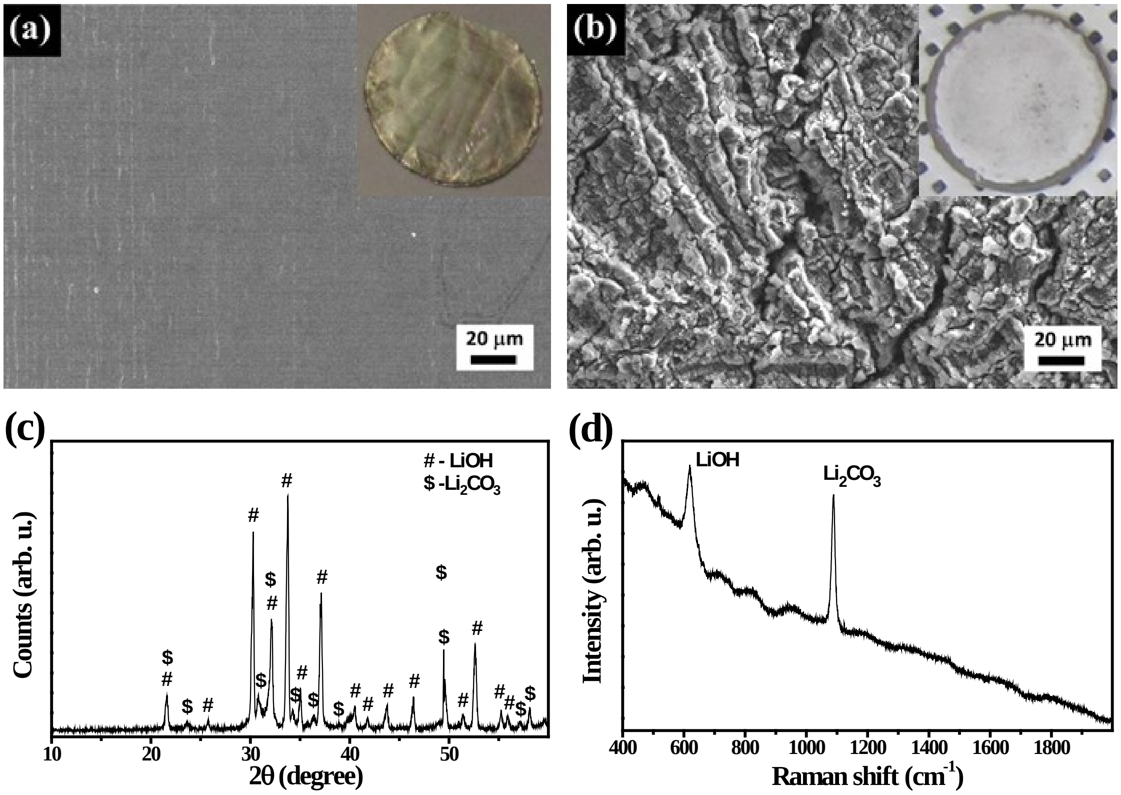

Fig. S12. FE-SEM images of Li anode; (a) fresh and (b) after testing (Inset: corresponding photograph images); examination of Li metal anode after testing (c) X-ray diffraction pattern and (c) Raman analysis.
